# CSW-YOLO: A traffic sign small target detection algorithm based on YOLOv8

**DOI:** 10.1371/journal.pone.0315334

**Published:** 2025-03-20

**Authors:** Qian Shen, Yi Li, YuXiang Zhang, Lei Zhang, ShiHao Liu, Jinhua Wu

**Affiliations:** 1 School of Automation, Huaiyin Institute of Technology, Huaian, Jiangsu, China; 2 Intelligent Energy Research Institute, Huaiyin Institute of Technology, Huaian, China; Monash University Malaysia, MALAYSIA

## Abstract

In order to improve the real-time and feasibility of traffic sign detection for autonomous driving in complex traffic environments, this paper proposes a small target detection algorithm for traffic signs based on the YOLOv8 model. First, the bottleneck of the C2f module in the original yolov8 network is replaced with the residual Faster-Block module in FasterNet, and then the new channel mixer convolution GLU (CGLU) in TransNeXt is combined with it to construct the C2f-faster-CGLU module, reducing the number of model parameters and computational load; Secondly, the SPPF module is combined with the large separable kernel attention (LSKA) to construct the SPPF-LSKA module, which greatly enhances the feature extraction ability of the model; Then, by adding a small target detection layer, the accuracy of small target detection such as traffic signs is greatly improved; Finally, the Inner-IoU and MPDIoU loss functions are integrated to construct WISE-Inner-MPDIoU, which replaces the original CIoU loss function, thereby improving the calculation accuracy. The model has been validated on two datasets Tsinghua-Tencent 100K (TT100K) and CSUST Chinese Traffic Sign Detection Benchmark 2021 (CCTSDB 2021), achieving Map50 of 89.8% and 98.9% respectively. The model achieves precision on par with existing mainstream algorithms, while being simpler, significantly reducing computational requirements, and being more suitable for small target detection tasks. The source code and test results of the models used in this study are available at https://github.com/lyzzzzyy/CSW-YOLO.git.

## Introduction

In recent years, with the gradual popularization of driverless technology, the speed and accuracy of traffic sign detection have become key factors in testing the safety of driverless vehicles. However, due to the complexity of actual road weather and surrounding environment, many model detection results are not ideal. Nowadays traffic sign target detection methods can be divided into two categories: traditional methods and deep learning methods.

Based on traditional methods, traffic signs are mainly detected by extracting color and shape features. For example, image segmentation is performed using color features such as red, blue, and yellow to initially locate areas that may contain traffic signs. Common color spaces include RGB, HSV, etc. [1]. Among them, HSV space is often used for color segmentation due to its robustness to changes in lighting.

Deep learning-based methods have achieved significant results in traffic sign detection. Such methods mainly include object detection based on candidate regions and object detection based on regression methods. R-CNN [2], Fast R-CNN [3] and Faster R-CNN [4] are representative of candidate region-based object detection methods. These methods first generate candidate regions using selective search or region proposal network RPN, then extract features from the candidate regions using convolutional neural network CNN, and complete classification in SVM or Softmax classifier. Faster R-CNN achieves fast generation of candidate regions by introducing RPN, greatly improving detection speed. In the feature extraction stage, multiple feature fusion strategies can be used, such as multi-scale feature fusion and multi-level feature fusion, to improve detection accuracy and robustness. Methods such as You Only Look Once(YOLO) and Single Shot MultiBox Detector(SSD) transform the object detection problem into a regression problem and directly predict the category and location of the target. They have the advantages of fast detection speed and good real-time performance.

In response to the above problems, this paper proposes a CSW-YOLO (CGLU-SPPF-LSKA- WISE-Inner-MPDIoU-YOLO) algorithm based on the YOLOv8s model for improvement, which can ensure both detection accuracy and simplicity of the model. The experimental results on multiple datasets ultimately verify its effectiveness. The contributions are as follows:

1) The residual module Faster-Block in FasterNet is used to replace the BottleNeck of the C2f module in the original yolov8 network, and the new channel mixer Convolutional GLU in the TransNeXt network is combined with the C2f module to construct a new C2f-Faster-CGLU module, achieving a significant reduction in model parameters and computational load.

2) Fusing the LSKA attention mechanism [5] with the SPPF module helps to better extract feature information from the feature map, thereby enhancing the detection performance of the model.

3) Optimizing the Head detection layer of the network by adding a small target detection layer greatly improves detection accuracy and makes the model more suitable for small target detection tasks such as traffic sign detection [6].

4) By integrating the Inner-IoU [7] loss function with the MPDIoU [8] loss function, a new loss function, WISE-Inner-MPDIoU, is constructed to improve the recall rate of the model, reduce loss, and further enhance the model’s detection accuracy.

## Related work

Currently, the common methods for traffic sign detection include those based on color, shape, multi-feature fusion, and deep learning. Among them, the method based on deep learning has demonstrated significant advantages. In terms of traffic sign recognition, commonly used methods include template matching, machine learning-based approaches, and deep learning-based methods. From the perspective of accuracy, deep learning-based traffic sign recognition methods achieve higher recognition rates. To cope with various environmental and weather conditions, researchers have designed a multi-path parallel fully convolutional neural network (CNN) to extract the color, shape, and texture features of traffic signs. This network structure is not only able to adapt to various environments, but also integrates shallow and deep features within the feature extraction network, ensuring accurate recognition of traffic signs with multi-scale variations. For instance, Sara et al. [9] and Ali [10] introduced a method that achieves rapid classification of traffic signs by extracting texture and color features from the RGB and HSV color model channels, and then fusing these features. Christian et al. [11] released the TUMTraf dataset, which contains multiple synchronized images including RGB images, allowing for detection based on the color features of the images. Hadi et al. [12] proposed a novel real-time road detection method that utilizes global modeling to generate images. These images are derived from RGB and HSV color values, which are computed separately and then integrated into the detection algorithm.

However, these traditional color and shape-based detection methods rely heavily on manual feature extraction, leading to issues such as low recognition accuracy, inaccurate results, and slow detection speeds. In contrast to these traditional detection methods, Ross et al. [13] combined region proposals with CNNs to obtain regions with CNN features.Wang et al. [14] proposed an improved Faster RCNN to detect small objects in traffic images, and employed post-processing of the model to enhance both accuracy and recall rates. Liang et al. [15] proposed an improved sparse R-CNN algorithm that integrates coordinate attention blocks with ResNeSt and constructs a feature pyramid to modify the backbone network, enabling the extracted features to focus on important information and improving detection accuracy. Cao et al. [16] proposed an improved sparse R-cnn model based on the original sparse R-cnn inspiration, by constructing hierarchical residual connections within each single base block of the original ResNest, enhancing the multi-scale representation ability of the backbone network, establishing a branch network, and adaptively recalibrating channel feature responses through global average pooling GAP operation, and establishing a fully connected layer, thereby achieving better accuracy and robustness. Wang et al. [17]introduced an end-to-end Generative Adversarial Network (GAN-STD) specifically designed for small object detection. This network enhances the similarity between small objects in shallow feature maps and large objects in deep feature maps, thereby reducing the representational disparity between small and large targets. You et al. [18] proposed a network algorithm based on lightweight SSD, which improves the real-time detection performance by replacing some of the 3×3 convolution kernels in the baseline network with 1×1 convolution kernels and removing some convolution layers.

Although methods such as R-CNN and SSD have achieved certain results in traffic sign detection, their processing speed and target detection accuracy are relatively backward compared to YOLO algorithm. Since Redmon first proposed the YOLO algorithm [19], it has developed rapidly in recent years due to its obvious advantages in detection accuracy and speed. Zhang et al. [20] proposed an improved model based on lightweight YOLOv5, which uses Ghost convolution and deep convolution to construct a new Bottleneck, and introduces BiFPN structure to reduce computation and parameters while enhancing feature fusion capabilities. Sun et al. [21] proposed an end-to-end framework LLTH-YOLOv5 specifically for low-light scenes, using GhostDarkNet53 in the Ghost module to replace the backbone network, enhancing the input image and improving detection performance. Du et al. [22] proposed a traffic sign detection method based on YOLOv8, introducing a spatial depth SPD module to increase the detection ability of objects, and using the WIoUv3 loss function to improve detection performance. He et al. [23] proposed a high-precision detection model, YOLOv8-CO, based on the YOLOv8 algorithm. By replacing C2f with C2fO, using global average pooling, and selecting CIoU, the accuracy is effectively improved. Xie et al. [24] proposed a new GRFS-YOLOv8 algorithm, which replaces the original SPPF with the GRF-SPPF module to capture richer multi-scale features from image feature mapping. A new SPAnet architecture was designed, and finally multiple GhostConv and C2fGhost were used to replace multiple CBS and C2f modules in the backbone and neck to achieve cost-effective operation speed and reduce model computation.

## The proposed method

### CSW-YOLO traffic sign detection algorithm

**Fig 1:**
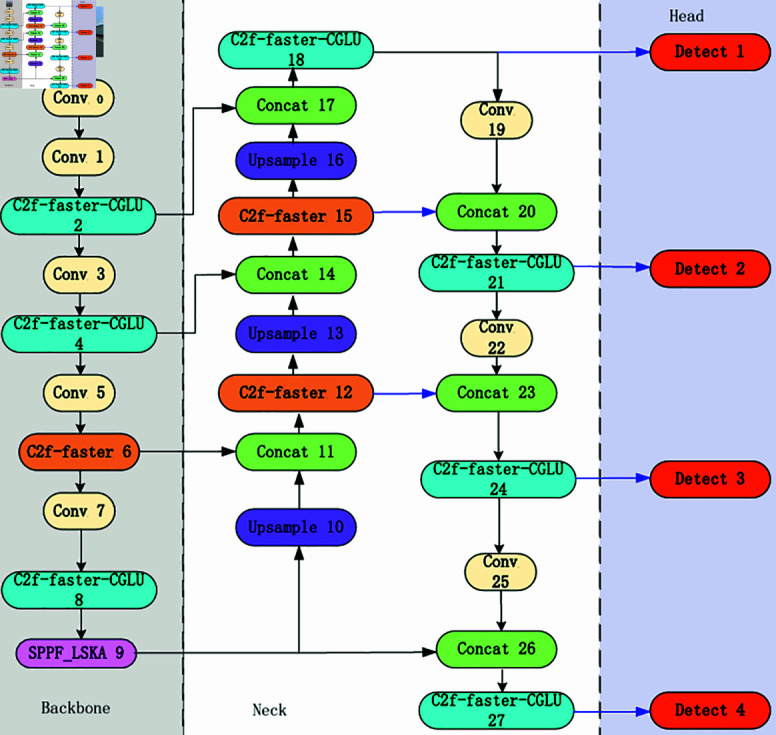
The improved CSW-YOLO model framework.

In the context of autonomous driving, traffic sign detection still faces many challenges. For example, the detection accuracy of algorithm models is not high, and the computational speed caused by excessive computation is not fast enough. Therefore, in order to ensure that vehicles have sufficient time to cope with complex traffic environments, it is necessary to improve the small target detection algorithm for traffic signs. In response to the above problems, this article proposes a new traffic sign detection algorithm model called CSW-YOLO, which uses C2f-Faster-CGLU to replace the C2f module of the original model, thereby significantly reducing the number of parameters and computational load of the model. The LSKA attention mechanism is added to SPPF module to improve the feature extraction ability of the model. When processing small target images such as traffic sign detection, a small target detection head is added, which greatly improves the detection accuracy. Finally, the scale factor ratio is introduced through Inner-IoU to control the size of the auxiliary bounding box, and it is effectively combined with the new boundary box similarity measurement MPDIoU loss function to further improve the model accuracy. The improved network structure diagram is shown in Fig 1.

### C2f-Faster-CGLU module

**Fig 2:**
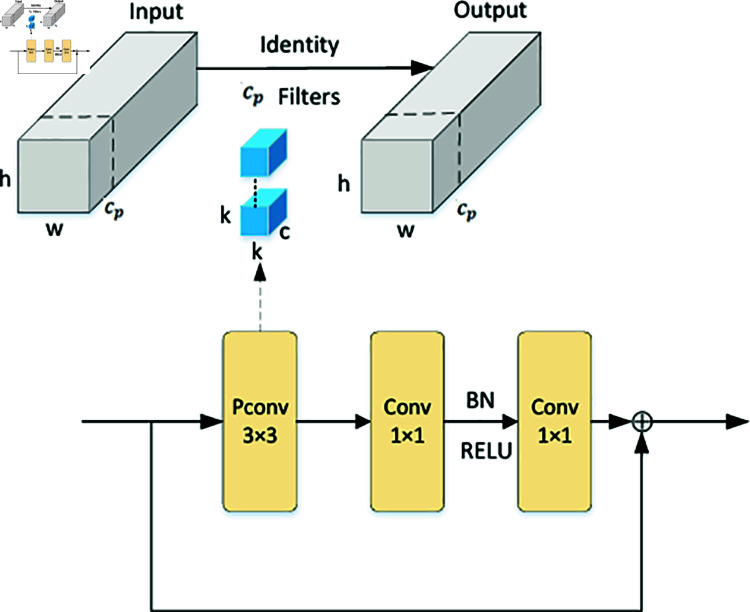
Block diagram of PConv and FasterNetBlock modules.

In 2023, Chen et al. [25] proposed a new fast neural networks (Faster Neural Networks), which maintains a high number of floating-point operations to a certain extent and proposes a new Partial Convolution (PConv) [26] convolution. The principle diagram is shown in Fig 2. It uses conventional convolution to extract features from a portion of the input channels, and treats the first channel as the entire feature map for computation while maintaining the number of channels unchanged. The number of partial channels is $c_{p}$. It can be considered that the input feature map and the output feature map have the same number of channels. The *FLOPs* of PConv can be expressed as:


$$\begin{array}{c} FLOPs=h\times w\times k^{2}\times c_{p}^{2} \end{array}$$ (1)

Among them, $c_{p}$ and c together constitute the separation ratio: $r=\frac{c_{p}}{c}$,When r=1/4, PConv has only 1/16 of the *FLOPs* of Conv, while PConv also has a smaller memory access:


$$\begin{array}{c} h\times w\times 2c_{p}+k^{2}\times c_{p}^{2}\approx h\times w\times 2c_{p} \end{array}$$ (2)

Replace the BottleNeck module in C2f with the FasterNetBlock module to obtain the C2f-Faster module. Thanks to PConv convolution, the FasterNetBlock has the advantages of faster speed and fewer parameters, while the loss of accuracy is limited. The BN module in the FasterNetBlock allows it to be combined with adjacent Conv modules to accelerate inference speed. This improvement can reduce the effect of computation and parameter quantity. The structure of the FasterNetBlock is shown in Fig 2.

**Fig 3:**
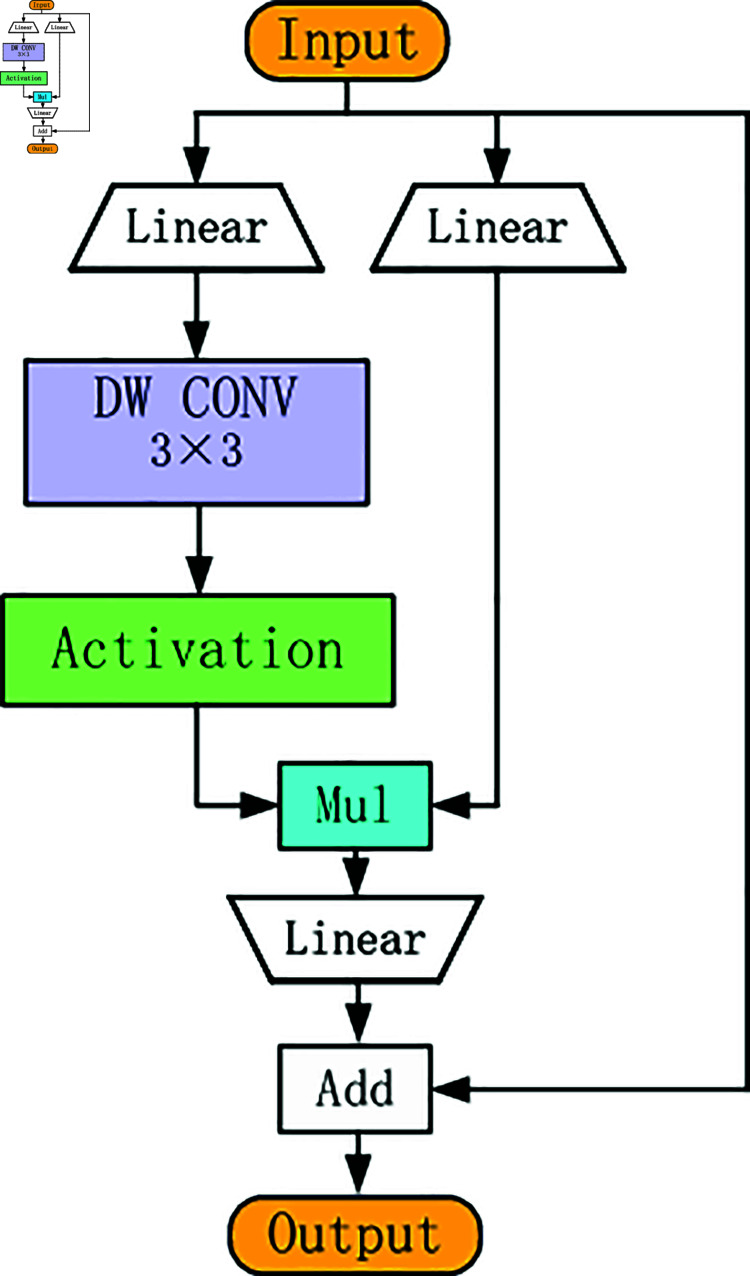
Structure diagram of the CGLU module.

In 2024, Dai et al. [27] proposed a hierarchical visual backbone network, TransNeXt, which combines aggregated attention as a token mixer and convolutional GLU as a channel mixer. This article utilizes the CGLU module and the C2f-Faster module to create a new C2f-Faster-CGLU module. The specific structure of the CGLU module is shown in Fig 3. Adding a minimum form of 3 × 3 deep convolution before the activation function of the GLU gating branch can make its structure conform to the design concept of gating channel attention, and transform it into a gating channel attention mechanism based on nearest neighbor features.

### SPPF-LSKA module

**Fig 4:**
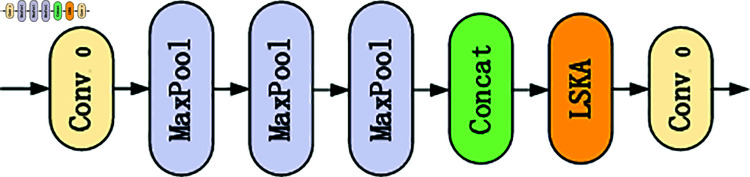
SPPF-LSKA module structure.

Due to the small target size of traffic sign detection and the complex detection environment, the LSKA attention mechanism is introduced into the SPPF module to construct the SPPF-LSKA module, as shown in Fig 4. This module can better extract feature information from the feature map to enhance the detection performance of the model. LSKA is an improvement on the LKA [28] (Large Kernel Attention) attention mechanism. The LKA module uses a large convolution kernel decomposition approach. Standard convolution can be decomposed into three parts: depth convolution, depth extension convolution, and point convolution. LKA decomposes the *K* × *K* convolution into a depth convolution output with a kernel size of  ( 2*d* − 1 ) × ( 2*d* − 1 ) , which is used to capture local spatial information. At the same time, the depth convolution compensates for subsequent depth extension convolution with a kernel size of $\left[\frac{k}{d}]\;\times \;[\frac{k}{d}\right]$, which is used to obtain global spatial information from the depth convolution output, and finally outputs through a 1 × 1 convolution.By decomposing the two-dimensional kernels of deep convolution and deep dilated convolution into two cascaded one-dimensional separable kernels, an equivalent and improved LKA structure can be obtained. This modified configuration is referred to as LSKA. While maintaining similar performance, LSKA modules are significantly reduced in terms of computational complexity and memory footprint. In addition, with the increase of core size, the proposed LSKA module focuses more on shape extraction than texture, which makes it possible to distinguish other features in complex textures.

### Addition of small target detection layer

In the field of traffic sign detection, the lack of obvious feature information in the target poses a challenge. In the design of the original YOLOV8 algorithm, the three detection layers adopt relatively large downsampling factors, which makes it more difficult for the algorithm to fully capture complex features of small targets. To address this issue, the neck and detection head parts were improved by adding a small target detection layer for extracting small features. The small target detection layer is located after the last few convolutional blocks of the backbone network and includes several additional convolutional layers for extracting more detailed features. This detection layer can output a feature map with a size of 160 × 160 pixels and can detect targets with a size of 4 × 4 pixels or larger. This method is more suitable for small target detection such as traffic signs.

### Improved loss function WISE-Inner-MPDIoU

In order to compensate for the weak generalization and slow convergence of existing IoU losses in different detection tasks, this paper proposes using the Wise-Inner-MPDIoU loss function to calculate the loss and accelerate the bounding box regression process. The specific steps are as follows: In response to the problems that traditional target detection or bounding box regression methods have when dealing with bounding box prediction, such as fixed scaling factors, lack of flexibility, and poor adaptability to extreme situations, in Inner-IoU, a proportional factor ratio is introduced to control the size of the auxiliary bounding box, and the MPDIoU loss function is introduced to solve the problem of not being able to optimize when the aspect ratio of the predicted bounding box is the same as the real bounding box, but the width and height values are completely different.

Typically, we use the coordinates of the upper left and lower right points to define a unique rectangle. Inspired by the geometric properties of bounding boxes, we design a iou-based MPDIoU metric, which is calculated by directly minimizing the distance between the predicted bounding box and the ground truth bounding box at the top left and bottom right points. It can comprehensively consider the overlapping area of the predicted pattern, the distance between the center points, and the deviation in width and height. $\left(x_{1}^{A},y_{1}^{A}\right)$and $\left(x_{2}^{A},y_{2}^{A}\right)$ represent the coordinates of the upper left and lower right points of A.$\left(x_{1}^{B},y_{1}^{B}\right)$and $\left(x_{2}^{B},y_{2}^{B}\right)$ represent the coordinates of the upper left and lower right points of B. *w* and *h* are the width and height of the prediction box, respectively. $d_{1}^{2}$ represents the square of the distance between the second rectangle and the upper left corner of the first rectangle, and $d_{2}^{2}$ represents the square of the distance between the second rectangle and the lower right corner of the first rectangle.


$$\begin{array}{c} d_{1}^{2}={\left(x_{1}^{B}-x_{1}^{A}\right)}^{2}+{\left(y_{1}^{B}-y_{1}^{A}\right)}^{2} \end{array}$$ (3)

$$\begin{array}{c} d_{2}^{2}={\left(x_{2}^{B}-x_{2}^{A}\right)}^{2}+{\left(y_{2}^{B}-y_{2}^{A}\right)}^{2} \end{array}$$ (4)

$$\begin{array}{c} MPDIoU=\frac{A\cap B}{A\cup B}-\frac{d_{1}^{2}}{w^{2}+h^{2}}-\frac{d_{2}^{2}}{w^{2}+h^{2}} \end{array}$$ (5)

The MPDIoU loss function formula is as follows:


$$\begin{array}{c} L_{MPDIoU}=1-MPDIoU=1+\frac{d_{1}^{2}}{d^{2}}+\frac{d_{2}^{2}}{d^{2}}-IoU \end{array}$$ (6)

The ground truth (GT) box and anchor are labeled as $b^{gt}$ and *b*, respectively. The center point of the GT box and the inner GT box is represented with $\left(x_{c}^{gt},y_{c}^{gt}\right)$. $\left(x_{c},y_{c}\right)$ represents the inner anchor and the center point of the anchor point. The width and height of the anchor are identified by *w* and *h*. $b_{l}^{gt}$, $b_{r}^{gt}$, $b_{t}^{gt}$, $b_{b}^{gt}$ represent the coordinates of the left, right, top and bottom boundaries of the ground truth bounding box, respectively. $b_{l}$, $b_{r}$, $b_{t}$, $b_{b}$ represent the coordinates of the left, right, top and bottom boundaries of the predicted bounding box, respectively. Inter is calculated by calculating the overlap of two bounding boxes on the x-axis and y-axis, while union is calculated by adding the areas of the two bounding boxes and then subtracting their intersection area. The formula for Inner-IoU is as follows:


$$\begin{array}{c} b_{l}^{gt}=x_{c}^{gt}-\frac{w^{gt}*ratio}{2},b_{r}^{gt}=x_{c}^{gt}+\frac{w^{gt}*ratio}{2} \end{array}$$ (7)

$$\begin{array}{c} b_{t}^{gt}=y_{c}^{gt}-\frac{h^{gt}*ratio}{2},b_{b}^{gt}=y_{c}^{gt}+\frac{h^{gt}*ratio}{2} \end{array}$$ (8)

$$\begin{array}{c} b_{l}=x_{c}-\frac{w*ratio}{2},b_{r}=x_{c}+\frac{w*ratio}{2} \end{array}$$ (9)

$$\begin{array}{c} b_{t}=y_{c}-\frac{h*ratio}{2},b_{b}=y_{c}+\frac{h*ratio}{2} \end{array}$$ (10)

$$\begin{array}{c} inter=\left(min(b_{r}^{g\iota },b_{r})-max(b_{l}^{g\iota },b_{l})\right)*\left(min(b_{b}^{gt},b_{b})-max(b_{t}^{gt},b_{t})\right) \end{array}$$ (11)

$$\begin{array}{c} union=(w^{gt}*h^{gt})*{\left(ratio\right)}^{2}+(w*h)*{\left(ratio\right)}^{2}-inter \end{array}$$ (12)

$$\begin{array}{c} IoU^{inner}=\frac{inter}{union} \end{array}$$ (13)

The loss function is as follows:


$$\begin{array}{c} L_{Inner-IoU}=1-IoU^{inner} \end{array}$$ (14)

This article combines Inner-IoU with MPDIoU to increase the accuracy of model detection. The loss function is as follows:


$$\begin{array}{c} L_{Inner-MPDIoU}=L_{MPDIoU}+IoU-IoU^{inner} \end{array}$$ (15)

## Experiment and discussion

The performance evaluation of the traffic sign detection model on the TT100K [29] and CCTSDB 2021 [30] datasets was conducted to further verify the superiority of the model by comparing it with other mainstream methods. The experiment uses the TT100K dataset produced by Tsinghua University as the base dataset, and conducts generalization experiments on the CCTSDB 2021 dataset.

### Introduction to the dataset

1) The TTT100K dataset contains a total of 221 categories, but there is a problem of uneven distribution of categories. To alleviate the problem of insufficient samples, we removed categories with fewer than 100 traffic signs, leaving 45 categories. The extracted images were divided into a training set and a test set, with 7227 preprocessed training images and 1899 preprocessed test images.

2) The CCTSDB 2021 dataset has been expanded based on the 2017 dataset, with more than 4,000 real traffic scene images added. It contains three main categories of traffic signs: mandatory, prohibited and warning. The final preprocessed training set includes 16,356 images and the test set includes 1,500 images. It includes various complex weather conditions, such as rain, snow, fog, etc.

### Experimental environment and evaluation indicators

The operating system of the experimental computer is 64-bit Windows11 Professional Edition, the GPU is NVIDIA GeForce RTX4060Ti, the video memory size is 16G, the CUDA version is 12.1, the deep learning framework Pytorch 2.1.0, and Python 3.11. The experimental training epoch is 300. The number of workers and the batch size is 8 and 16. The input photo size is 640, and the initial learning rate is 0.01.

The samples of the target detection results can be roughly divided into three categories. True positive (TP) indicates the detection of the correct target, false negative (FN) indicates the undetected target, and false positive (FP) indicates the detection of the wrong target. This experiment mainly uses mAP [31] (mean average precision) to evaluate performance.

mAP is a commonly used metric for evaluating object detectors, which quantifies the accuracy and recall of object detectors at different intersections exceeding a joint threshold to measure their accuracy. IoU measures the overlap between the predicted box and the actual true box.


$$\begin{array}{c} AP=\underset{n}{\sum }(R_{n}-R_{n-1})P_{n} \end{array}$$ (16)

$$\begin{array}{c} mAP=\frac{1}{N}\overset{N}{\underset{i=1}{\sum }}AP_{i} \end{array}$$ (17)

In addition, the speed and efficiency of the target detector are measured by comparing the number of parameters and GFLOPs of the model. The lower the values of the first two indicators, the higher the efficiency of the model.

### Loss function comparison test

In this section, in order to demonstrate the advantage of Wise-Inner-MPDIoU with a loss function ratio of 0.7, we conducted comparative experiments on Wise-Inner-MPDIoU with SIoU, CIoU, EIoU, GIoU, WIoU and other typical loss functions using the TT100K dataset. The experimental results are summarized in Table 1. Compared to several other loss functions, the Wise-Inner-MPDIoU loss function shows a higher mAP50 value. This indicates that the model has good generalization ability to adapt to small target detection tasks such as traffic sign detection.

**Table 1:** Comparison of different loss functions.

*Loss* *Functions*	mAP50(%)
*CIoU*	88.4
*EIoU *	88.7
*SIoU*	87.3
*GIoU *	87.5
*WIoU*	89.1
*MPDIoU *	88.6
*Wise*–*Inner*–*MPDIoU*	89.8

### Ablation experiment

This section conducts ablation experiments on the TT100K dataset to verify the impact of model components on performance. YOLOv8 is selected as the base model, and comparisons are made using Params, GFLOPs, and mAP50 as metrics. Compared to the base model, mAP50 improves by 4.9%, while reducing the number of parameters by 2.45M while maintaining the same computational load. Therefore, the model significantly improves detection accuracy while maintaining the speed of model algorithm operations.

**Fig 5:**
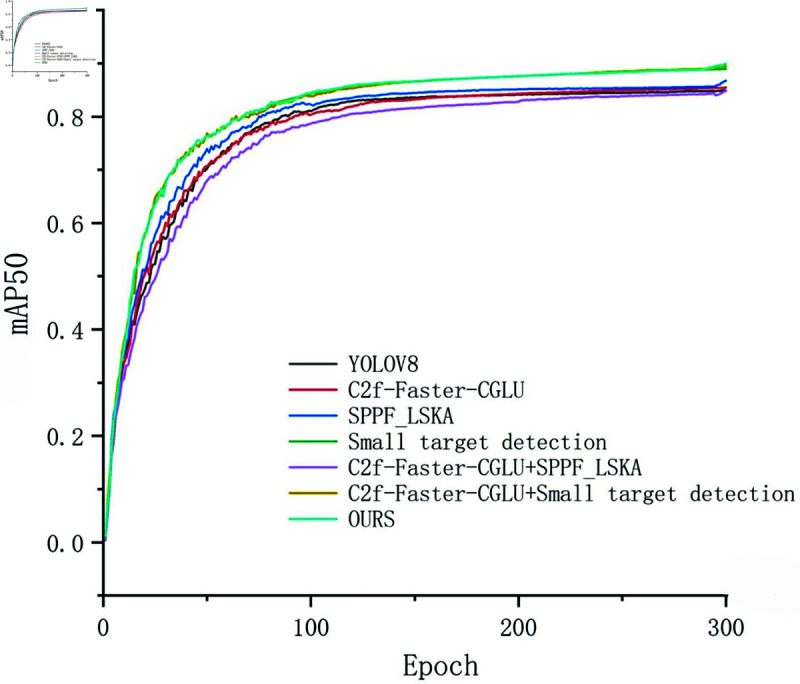
The detection accuracy of each ablation experiment.

The modules used in this paper have all improved the overall performance of the model. Compared to the YOLOv8 base model, when using the improved C2f-Faster-CGLU module, the number of parameters and computational load of the model are reduced by 2.25M and 7.4 GFLOPs, respectively, while slightly increasing accuracy. This indicates that the new C2f-Faster-CGLU ensures the lightweight nature of the model without losing too many features, and can still guarantee the accuracy of model detection. When using the SPPF-LSKA module, it can be found that although the number of parameters and computational load increase slightly, the accuracy of feature extraction improves. Then tried to increase the detection of small objects and found that although the computational load increased by nearly 10 GFLOPs, the accuracy improved by 4%, significantly enhancing the detection accuracy of the model. Next, we conducted permutation and combination experiments of various modules. First, we combined the C2f-Faster-CGLU and SPPF-LSKA modules. The results show that under the influence of the lightweight module C2f-Faster-CGLU, the increase in the number of parameters and computation caused by the SPPF-LSKA module is greatly improved; Then, C2f-Faster-CGLU was combined with the small target detection module. The results showed that this combination ensured a relatively stable number of parameters and computational load, while greatly improving the accuracy of fine measurement. The results are shown in Table 2. At this point, the model in this paper has achieved the best results. The following Fig 5 shows the detection accuracy of each ablation experiment, in order to more intuitively see the differences.

**Table 2:** Ablation experiment of TT100K dataset.

*C*2*f *–*faster*–*CGLU*	SPPF-LSKA	Small target detection	Wise-Inner-MPDIoU	mAP50(%)	Params(M)	GFLOPs
			*✓*	84.9	11.15	28.7
*✓*			*✓*	85.4	8.9	21.3
	*✓*		*✓*	86.7	12.2	29.4
		*✓*	*✓*	88.9	10.9	38.3
*✓*	*✓*		*✓*	86.2	9.0	21.5
*✓*		*✓*	*✓*	89.4	7.6	28.8
*✓*	*✓*	*✓*	*✓*	89.8	8.7	29.7

**Fig 6:**
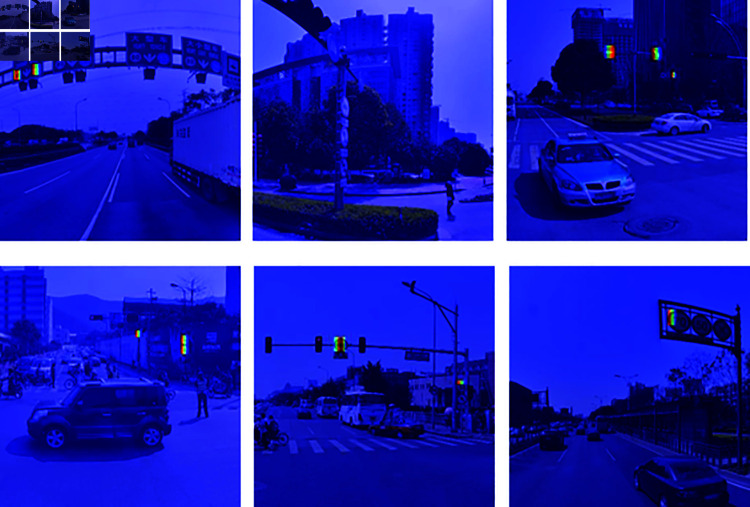
Visualization result of yolov8 heatmap.

**Fig 7:**
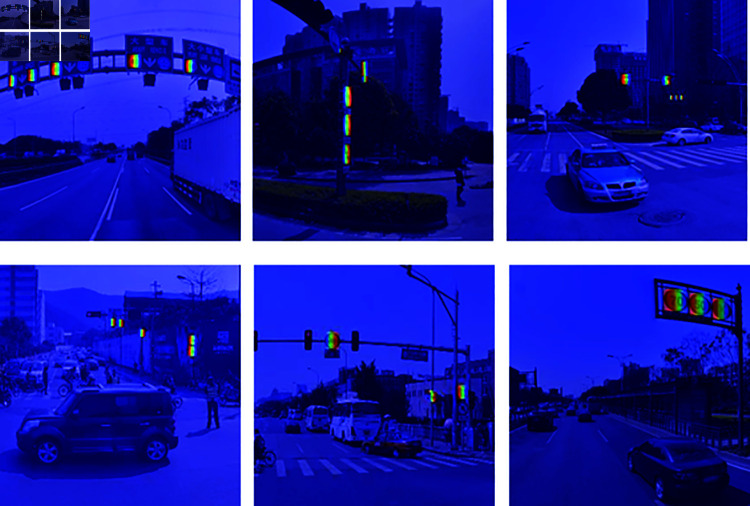
Thermodynamic diagram visualization results after adding the LSKA attention mechanism.

To demonstrate the superior performance of the LSKA attention mechanism in this model, we used the GradCam heatmap visualization method to compare the YOLOv8 baseline model with the model with the LSKA attention mechanism added. As can be seen from Figs 6 and 7, the model with the LSKA attention mechanism added is better able to detect small target tasks such as traffic signs and avoiding many cases of missed detections.

#### Experimental results on the TT100K dataset.

To verify the effectiveness of the CSW-YOLO model, we reproduced the detection structures of various mainstream object detection networks on the enhanced TT100K and compared them with YOLOv8s and our model. The comparison results can refer to Table 3, where it can be clearly seen that better results have been achieved in terms of Params, GFLOPs and mAP50.

**Table 3:** Comparison with other models on the TT100K dataset.

*Module*	mAP50(%)	Params(M)	GFLOPs
*FasterR*–*CNN*	56.3	41.6	85.0
*YOLOv*5*s*	71.4	7.1	16.3
*YOLOv*6*s*	83.7	17.2	44.1
*YOLOv*7*s*	58.3	37.4	105.8
*YOLOv*8*s*	84.9	11.15	28.7
*SC*–*YOLO*[32]	90.4	6.1	44.3
*ETSR*–*YOLO*[33]	88.2	7.5	37.6
*Yolov*8*s* + *spd* + *sk* + *Wiou* [ 22 ]	90.6	8.8	65.7
*GRFS*–*YOLOv*8[34]	80.3	1.71	10.9
*CSW *–*YOLO*(*Ours*)	89.8	8.7	29.7

As the results in Table 3, it can be seen that both SC-YOLO and Yolov8s+spd+sk+Wiou models achieve high detection accuracy, but the model CSW-YOLO in this paper greatly reduces the computational load while achieving comparable accuracy. Compared with GRFS YOLOv8, although GRFS YOLOv8 model has extremely small parameter and computational load, and is extremely lightweight, its detection accuracy is only 71.2%. Compared with the classic two-stage algorithm Faster R-CNN, both Map50 and Params show significant improvements for this model.

#### Experimental results on the CCTSDB 2021 dataset.

To verify the effectiveness of the model in this paper, this section uses the CSW-YOLO model to conduct a generalization experiment on another open-source traffic sign dataset CCTSDB 2021, and compares its experimental results with those of different mainstream algorithms. The results are shown in Table 4.

**Table 4:** Comparison with other models on the CCTSDB 2021 dataset.

*Module*	Map50(%)	Params(M)	GFLOPs
*FasterR*–*CNN*	77.4	41.6	85.0
*YOLOv*5*s*	96.4	7.1	16.3
*YOLOv*8*s*	98.2	11.15	28.7
*SC*–*YOLO*[32]	84.3	6.1	44.3
*ETSR*–*YOLO*[33]	98.3	7.5	37.6
*Yolov*8*s* + *spd* + *sk* + *Wiou* [ 22 ]	86.5	8.8	65.7
*GRFS*–*YOLOv*8[34]	80.3	1.71	10.9
*CSW *–*YOLO*(*Ours*)	98.9	8.7	29.7

As in Table 4, it can be concluded that the model in this article has achieved an accuracy of 98.9%, which is a great breakthrough in detection accuracy compared to other mainstream models. This further proves the superior performance of the model and also demonstrates the strong generalization ability of the model algorithm.

#### Visualization.

**Fig 8:**
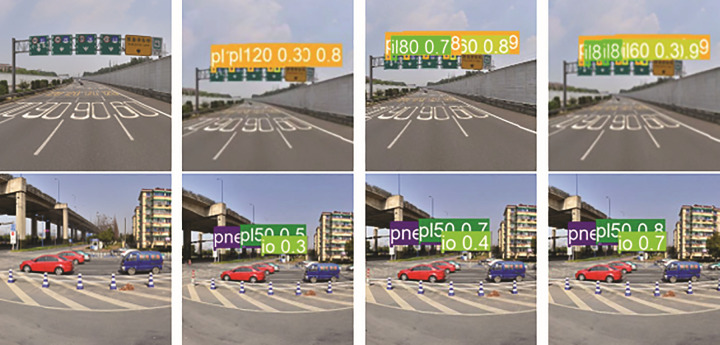
Traffic sign detection results of TT100K.

**Fig 9:**
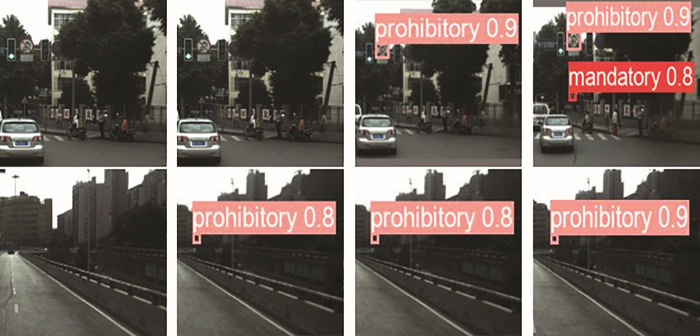
Traffic sign detection results of CCTSDB 2021.

In this section, in order to demonstrate the detection capability of the CSW-YOLO model, the detection results under different categories of data sets are listed, as shown in Figs 8 and 9. The first, second, third, and fourth images are the original image, the YOLOv5 result image, the YOLOv8 result image, and the CSW-YOLO result image, respectively.

As can be seen from Fig 8, the CSW-YOLO model has a higher recall rate than the YOLOv5 and YOLOv8 models. In the TT100K dataset, YOLOv5 has serious missed detection and low detection accuracy. YOLOv8 also had missed detections, while the CSW-YOLO model could detect both label types. According to the CCTSDB2021 dataset, it can be seen from Fig 9 that YOLOv5 has a missing detection phenomenon for both labels, and YOLOv8 has a missing detection for mandatory. However, the model in this article can detect it well with high confidence. In summary, the CSW-YOLO model proposed in this paper has better detection performance and is more suitable for small target detection tasks such as traffic sign detection.

## Conclusion

In order to improve the real-time and feasibility of traffic sign detection for autonomous driving in complex traffic environments, this paper proposes a traffic sign detection algorithm CSW-YOLO for autonomous driving. In order to ensure the computational speed of the model, the residual module Faster-Block in FasterNet and the new channel mixer Convolutional GLU in TransNeXt network are combined. Subsequently, the LSKA attention mechanism is used to improve the feature extraction ability of the SPPF to enhance the model. Following that, the addition of a small target detection layer makes the model more suitable for small target detection tasks such as traffic signs. Finally, the accuracy is further improved by improving the WISE-Inner-MPDIoU loss function. Compared with other models, the CSW-YOLO model greatly improves the detection accuracy while ensuring the computational speed, making it more suitable for practical applications in autonomous driving in the future.

Although the model has high detection accuracy, it still has shortcomings. The model only detects traffic signs under normal weather conditions and does not generalize to other severe weather conditions. Therefore, in future experiments, we need to further improve the detection accuracy while expanding the data set to achieve detection under severe weather conditions as much as possible, and further improve the robustness and generalization ability of the model.

## Supporting information

S1 Data All Data is stored at https://github.com/lyzzzzyy/CSW-YOLO.git.
(ZIP)
