# Supplementary figures and images for: CSW-YOLO: A traffic sign small target detection algorithm based on YOLOv8
Source: PLoS One. 2025 Mar 20;20(3):e0315334. doi: 10.1371/journal.pone.0315334 (PMC11925293; doi:10.1371/journal.pone.0315334)

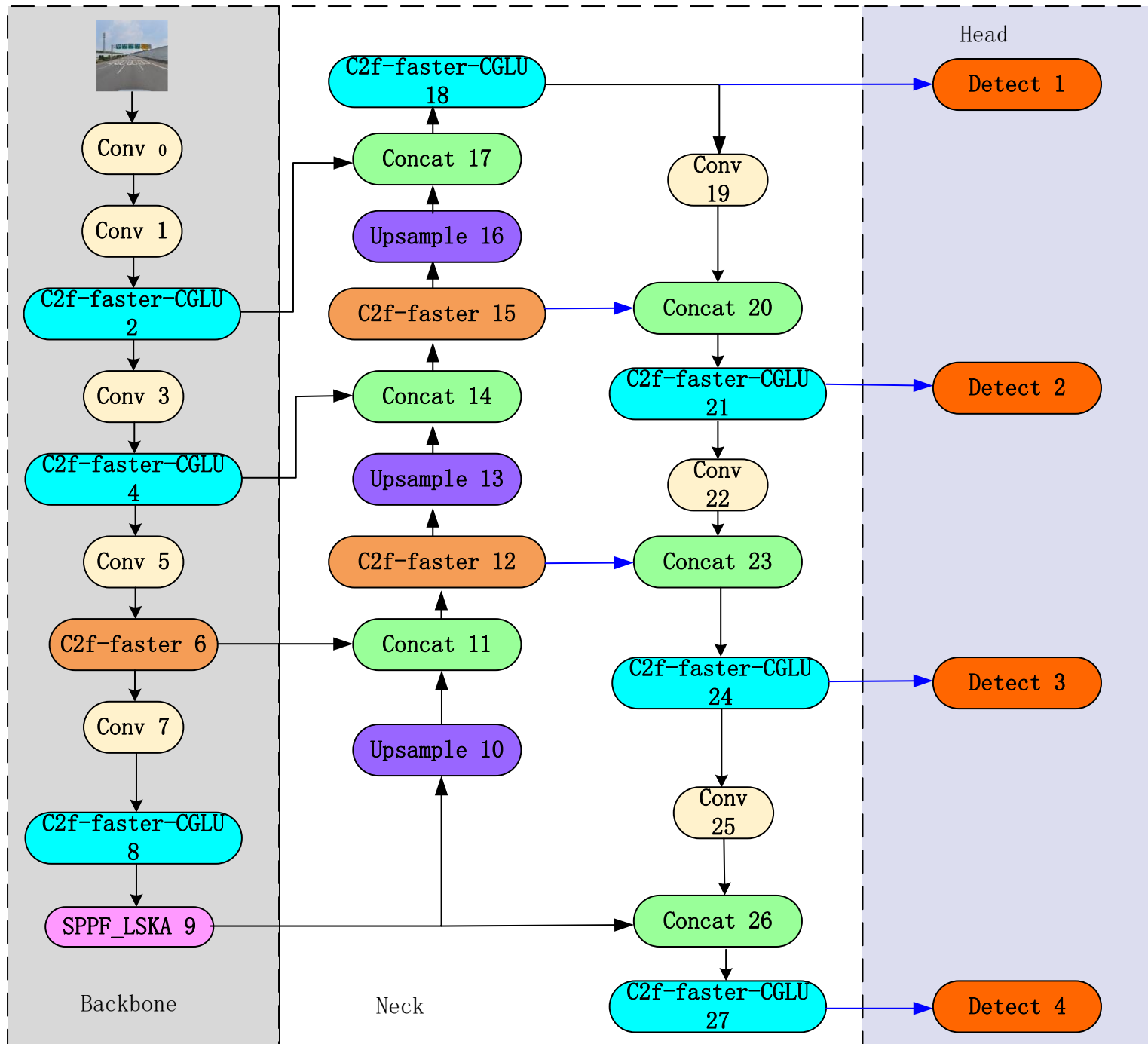

Supplement: S1 Data — All Data is stored at https://github.com/lyzzzzyy/CSW-YOLO.git. (ZIP) [file pone.0315334.s001.zip › Fig/Fig 1.pdf]

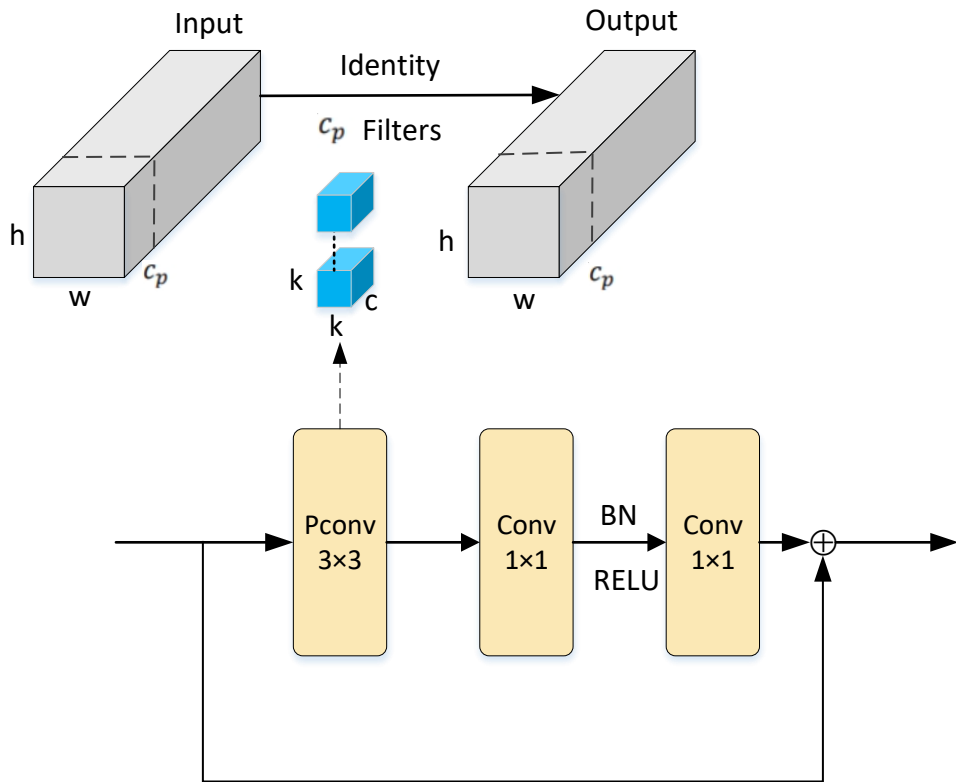

Supplement: S1 Data — All Data is stored at https://github.com/lyzzzzyy/CSW-YOLO.git. (ZIP) [file pone.0315334.s001.zip › Fig/Fig 2.pdf]

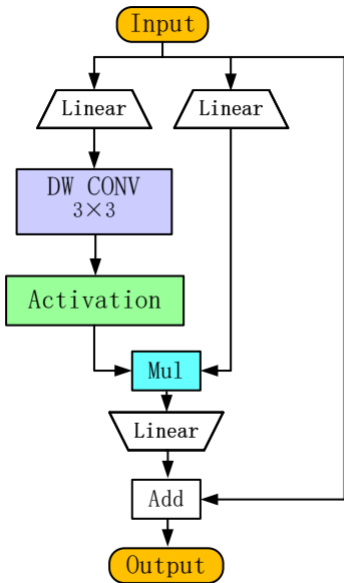

Supplement: S1 Data — All Data is stored at https://github.com/lyzzzzyy/CSW-YOLO.git. (ZIP) [file pone.0315334.s001.zip › Fig/Fig 3.pdf]

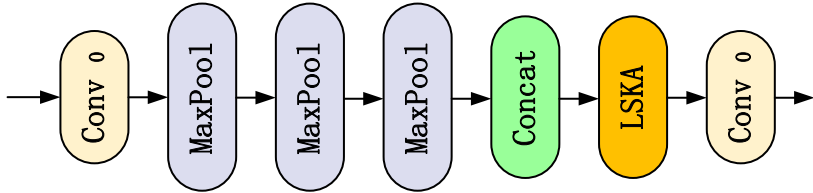

Supplement: S1 Data — All Data is stored at https://github.com/lyzzzzyy/CSW-YOLO.git. (ZIP) [file pone.0315334.s001.zip › Fig/Fig 4.pdf]

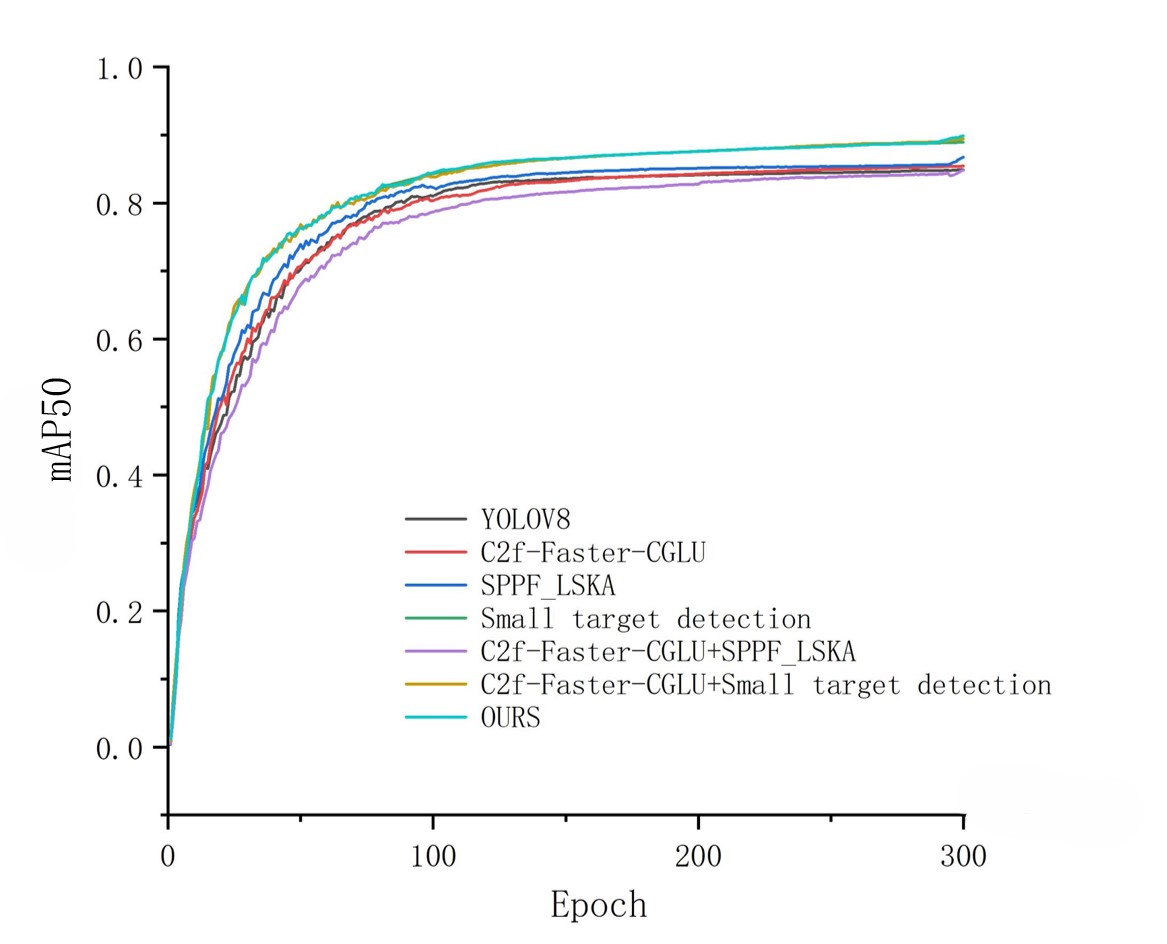

Supplement: S1 Data — All Data is stored at https://github.com/lyzzzzyy/CSW-YOLO.git. (ZIP) [file pone.0315334.s001.zip › Fig/Fig 5.jpg]

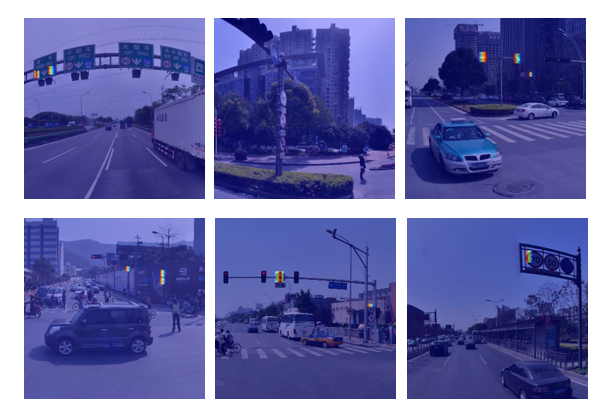

Supplement: S1 Data — All Data is stored at https://github.com/lyzzzzyy/CSW-YOLO.git. (ZIP) [file pone.0315334.s001.zip › Fig/Fig 6.jpg]

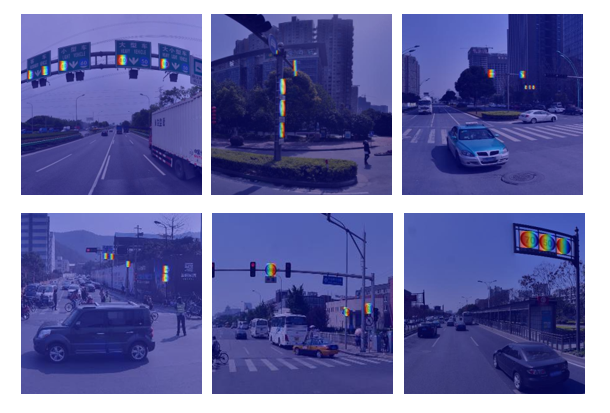

Supplement: S1 Data — All Data is stored at https://github.com/lyzzzzyy/CSW-YOLO.git. (ZIP) [file pone.0315334.s001.zip › Fig/Fig 7.jpg]

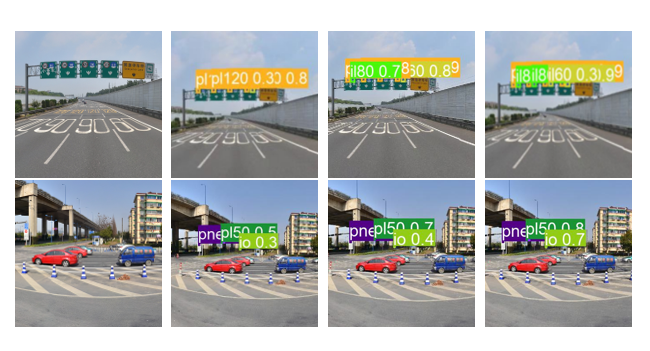

Supplement: S1 Data — All Data is stored at https://github.com/lyzzzzyy/CSW-YOLO.git. (ZIP) [file pone.0315334.s001.zip › Fig/Fig 8.jpg]

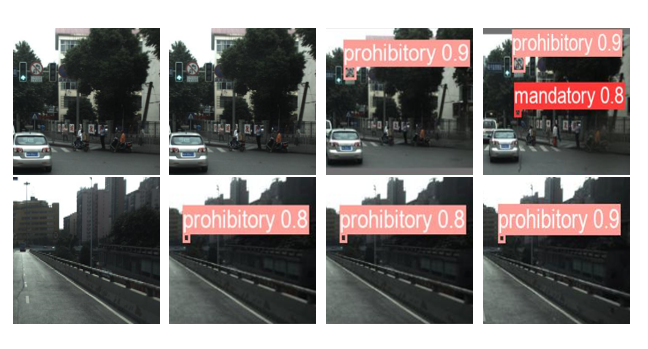

Supplement: S1 Data — All Data is stored at https://github.com/lyzzzzyy/CSW-YOLO.git. (ZIP) [file pone.0315334.s001.zip › Fig/Fig 9.jpg]
